# Effect of lordosis on adjacent levels after lumbar interbody fusion, before and after removal of the spinal fixator: a finite element analysis

**DOI:** 10.1186/s12891-019-2886-4

**Published:** 2019-10-25

**Authors:** Fon-Yih Tsuang, Jui-Chang Tsai, Dar-Ming Lai

**Affiliations:** 1 Division of Neurosurgery, Department of Surgery, National Taiwan University Hospital, Taipei, Taiwan; 2 Department of Traumatology, National Taiwan University Hospital, Taipei, Taiwan; 3 Center for Optoelectronic Biomedicine, National Taiwan University College of Medicine, Taipei, Taiwan

**Keywords:** posterior lumbar fusion, Finite element analysis, adjacent-segment disease, Spinal fixator, lumbar lordosis

## Abstract

**Background:**

Literature indicates that adjacent-segment diseases after posterior lumbar interbody fusion with pedicle screw fixation accelerate degenerative changes at unfused adjacent segments due to the increased motion and intervertebral stress. Sagittal alignment of the spine is an important consideration as achieving proper lordosis could improve the outcome of spinal fusion and avoid the risk of adjacent segment diseases. Therefore, restoration of adequate lumbar lordosis is considered as a major factor in the long-term success of lumbar fusion. This study hypothesized that the removal of internal fixation devices in segments that have already fused together could reduce stress at the disc at adjacent segments, particularly in patients with inadequate lordosis. The purpose of this study was to analyze the biomechanical characteristics of a single fusion model (posterior lumbar interbody fusion with internal fixation) with different lordosis angles before and after removal of the internal fixation device.

**Methods:**

Five finite element models were constructed for analysis; 1) Intact lumbar spine without any implants (INT), 2) Lumbar spine implanted with a spinal fixator and lordotic intervertebral cage at L4-L5 (FUS-f-5c), 3) Lumbar spine after removal of the spinal fixator (FUS-5c), 4) Lumbar spine implanted with a spinal fixator and non-lordotic intervertebral cage at L4-L5 (FUS-f-0c), and 5) Lumbar spine after removal of the spinal fixator from the FUS-f-0c model (FUS-0c).

**Results:**

The ROM of adjacent segments in the FUS-f-0c model was found to be greater than in the FUS-f-5c model. After removing the fixator, the adjacent segments in the FUS-5c and FUS-0c models had a ROM that was similar to the intact spine under all loading conditions. Removing the fixator also reduced the contact forces on adjacent facet joints and reduced the peak stresses on the discs at adjacent levels. The greatest increase in stress on the discs was found in the FUS-f-0c model (at both L2/L3 and L3/L4), with intervertebral stress at L3/L4 increasing by 83% when placed in flexion.

**Conclusions:**

This study demonstrated how removing the spinal fixation construct after bone fusion could reduce intradiscal pressure and facet contact forces at adjacent segments, while retaining a suitable level of lumbar lordosis.

## Background

The use of internal fixation devices combined with an interbody cage is common in spinal fusion procedures and has been demonstrated to significantly improve the fusion rate [1, 2]. Although the benefits and clinical outcomes have been widely reported, the fused region often succumbs to post-surgical adjacent-segment disease [3–5]. In 2018, Okuda et al. indicated the incidence of adjacent-segment diseases after posterior lumbar interbody fusion with pedicle screw fixation to be up to 9% at an average follow-up of 8.3 years, and the predicted survivorship of the adjacent segments fell by almost 90% at 10 years [6]. The increased motion and intervertebral stress at adjacent segments have been suggested as major factors in accelerating degenerative changes in unfused adjacent segments [7–9]. Using finite element analysis, Chen CS [10] and Hsieh YY et al. [11] demonstrated how the motion segment places additional stresses on the upper disc adjacent to the interbody fusion site. Serious symptomatic degenerative changes at the adjacent segments usually require additional decompression with fusion, but the quality of life and range of motion of patients are often impacted by such secondary interventions.

The likelihood of developing adjacent segment disease is influenced by a number of factors, including age, gender, etiology, fusion level and site, presence of an interbody cage, sagittal alignment, and the use of rigid pedicle screw instrumentation [12]. Sagittal alignment of the spine is an important surgical consideration because achieving proper lordosis could improve the outcome of spinal fusion and reduce the risk of adjacent segment diseases [13]. Restoration of adequate lumbar lordosis is considered a major factor for the long-term success of lumbar fusion. A cadaveric study has shown that hypolordosis at the instrumented segments increases shear forces in the upper adjacent level [9]. A finite element study by Zhao et al. [14] noted an increase in stress on the adjacent disc and decrease in spinal lordosis in patients who underwent interbody fusion with pedicle screw instrumentation. Unfortunately, failure to maintain or correct lumbar lordosis after fusion is common [15], and the management of a loss of lordosis in patients who undergo interbody fusion is still a challenge for surgeons.

In order to preserve the range of motion of the fusion site and decrease instrument related pain and metal hypersensitivity, this current study investigated the effects of removing all posterior instruments after complete solid fusion has occurred. Similarly, Hsieh et al. [11] suggested that removal of internal fixation devices after solid fusion could decrease the stress at adjacent segments. The authors hypothesized that the removal of internal fixation devices after fusion had occurred could provide major benefits to the patients by reducing stress at the disc at adjacent segments, especially in patients suffering from a loss of lordosis. The purpose of this study was to analyze the biomechanical characteristics of a single fusion model (posterior lumbar interbody fusion with internal fixation) with different lordosis angles before and after removal of the internal fixation device.

## Materials and methods

**Fig. 1:**
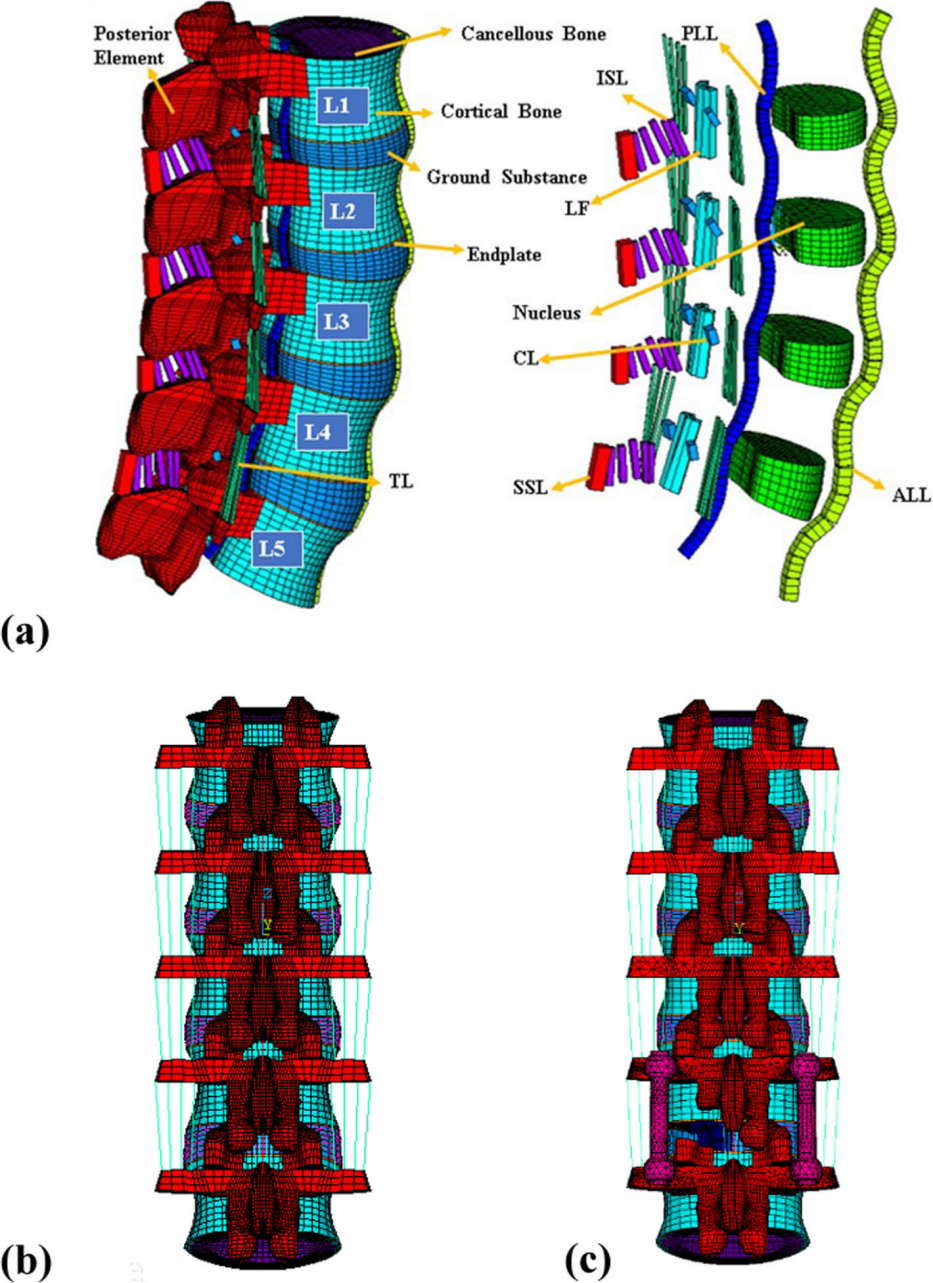
FE models of the spine with a spinal fixator and with the fixator removed; **a**) Bones, intervertebral discs, and ligaments of the intact spine. **b**) Mesh of intact FE models used in this study. **c**) The fusion and fixation model, with the L4–L5 segment immobilized by a posterior spinal fixator and fused by a stand-alone cage placed with the posterior corner

A finite element model of 5-level intact lumbar spine was created using the software ANSYS (ANSYS Inc., Canonsburg, PA, USA). Details of model validation, material properties and convergency testing are included in a previous study [16–18]. Briefly, Fig. 1**a** illustrates the complete lumbar model including vertebrae (L1-L5), intervertebral discs (IVDs) and seven ligaments. The IVDs are composed of an annulus fibrosus and nucleus pulposus, with the ground substance embedded with 12 double-crosslinked fiber layers. The annulus fibrosus was considered as an incompressible and hyperelastic material modeled using a 2-parameter (C1, C2) Mooney-Rivlin formulation, while the nucleus pulposus was considered as an incompressible fluid.

The CB PROT II Posterior Spinal System (Chin Bone Corp., Taiwan; US FDA 510(k): K142655) was used in this study, which is composed of titanium allow screws of diameter 5.5 mm connected by titanium rods. The intervertebral cage was modeled based on a stand-alone PEEK cage (Wiltrom, Taiwan) [11] and was implanted into the lumbar spine using an approach mimicking posterior lumbar interbody fusion (Fig. 1**b**). All components of each implant were modeled using 8-node solid elements.

Five finite element models were developed in this study:

1. INT: Intact lumbar spine without any implants (INT).
2. FUS-f-5c: INT implanted with an intervertebral cage at a lordotic angle of 5° and posterior spinal fixator (CB PROT II) at L4-L5 to fuse the L4-L5 segment.
3. FUS-5c: Posterior spinal fixator removed from the FUS-f-5c model.
4. FUS-f-0c: INT implanted with an intervertebral cage at a neutral angle (0°) and posterior spinal fixator (CB PROT II) at L4-L5 to fuse the L4-L5 segment (FUS-f-0c) without reconstructing the lordotic curvature.
5. FUS-0c: Posterior spinal fixator removed from the FUS-f-0c model.

For all fusion FE models (FUS-f-5c, FUS-5c, FUS-f-0c, and FUS-0c), the nucleus pulposus was removed and replaced by a cage and bone grafts. The interfaces between facet articular surfaces were treated as standard contact pairs at all levels. In order to simulate bone fusion, the interfaces between the endplate, cage and bone grafts were bonded in all fusion models, and the models were rigidly fixed at the base surface of the fifth lumbar vertebra. At the fused segment, two adjacent vertebrae were bridged using the CB PROT II system and a cage implanted at the IVD as detailed above. A hybrid multidirectional test method developed by Panjabi [19] was used to assess the effect of implantation on the levels adjacent to the fusion segment. The upper surface of the first lumbar vertebra was first loaded with a 150 N axial load, and then subjected to a pure unconstrained moment. The moment was increased in increments of 0.36 Nm until the ROM of the model (L1-L5) achieved 19° in flexion, 10° in extension, 10° in left torsion, and 20° in left lateral bending. The resultant ROM of each level from all lumbar models is detailed in Table 1.

**Table 1:** ROM of FE models at all motion segments

Motion	Model	L1-L2 (Degree)	L2-L3 (Degree)	L3-L4 (Degree)	L4-L5 (Degree)
Flexion	INT	4.45	4.43	4.34	5.78
		100%	100%	100%	100%
	FUS-f-5c	5.67	5.66	6.83	0.85
		127%	128%	157%	15%
	FUS-f-0c	5.70	5.72	7.25	0.33
		128%	129%	167%	6%
	FUS-5c	5.33	5.35	6.38	2.01
		120%	121%	147%	35%
	FUS-0c	5.56	5.55	6.87	1.09
		125%	125%	158%	19%
Extension	INT	3.05	2.62	2.56	2.57
		100%	100%	100%	100%
	FUS-f-5c	3.65	3.13	3.23	0.79
		120%	119%	126%	31%
	FUS-f-0c	3.70	3.21	3.48	0.44
		121%	123%	136%	17%
	FUS-5c	3.31	2.99	3.03	1.50
		109%	114%	118%	58%
	FUS-0c	3.49	3.11	3.19	1.11
		114%	119%	125%	43%
Lateral Bending	INT	5.74	5.01	4.7	4.48
		100%	100%	100%	100%
	FUS-f-5c	8.62	5.58	5.23	0.57
		150%	111%	111%	13%
	FUS-f-0c	8.72	5.61	5.28	0.39
		152%	112%	112%	9%
	FUS-5c	8.02	5.39	5.01	1.58
		140%	108%	107%	35%
	FUS-0c	8.02	5.53	5.19	1.26
		140%	110%	110%	28%
Torsion	INT	2.01	2.3	2.68	3.75
		100%	100%	100%	100%
	FUS-f-5c	4.91	2.26	2.59	0.99
		244%	98%	97%	26%
	FUS-f-0c	5.24	2.27	2.63	0.61
		261%	99%	98%	16%
	FUS-5c	4.41	1.99	2.34	2.01
		219%	87%	87%	54%
	FUS-0c	4.61	2.14	2.48	1.52
		229%	93%	93%	41%

The percentages indicate the ROM of all models normalized by the ROM of INT

This study investigated lumbar motion and stress, the results presented in Tables 1, 2 and 3 include the ROM of each motion segment, facet contact forces and peak disc stresses at L2–3 under flexion, extension, torsion, and left lateral bending.

**Table 2:** Facet joint forces in cephalic adjacent levels

Motion	Model	L2-L3	L3-L4
		(N)	(N)
Extension	INT	65	71
		100%	100%
	FUS-f-5c	82	105
		126%	148%
	FUS-f-0c	84	107
		129%	151%
	FUS-5c	73	90
		112%	127%
	FUS-0c	75	94
		115%	132%
Lateral Bending	INT	19	9
		100%	100%
	FUS-f-5c	23	17
		121%	189%
	FUS-f-0c	23	18
		121%	200%
	FUS-5c	21	14
		111%	156%
	FUS-0c	21	15
		111%	167%
Torsion	INT	125	124
		100%	100%
	FUS-f-5c	137	165
		110%	133%
	FUS-f-0c	137	168
		110%	135%
	FUS-5c	129	141
		103%	114%
	FUS-0c	129	141
		103%	114%

The percentages indicate the facet joint forces of all models normalized by the facet joint forces of INT

**Table 3:** Disc stresses at cephalic adjacent levels

Motion	Model	L2-L3	L3-L4
		(KPa)	(KPa)
Flexion	INT	880	742
		100%	100%
	FUS-f-5c	1150	1160
		131%	156%
	FUS-f-0c	1229	1361
		140%	183%
	FUS-5c	1079	1125
		123%	152%
	FUS-0c	1186	1241
		135%	167%
Extension	INT	398	424
		100%	100%
	FUS-f-5c	460	524
		116%	124%
	FUS-f-0c	467	533
		117%	126%
	FUS-5c	459	522
		115%	123%
	FUS-0c	460	523
		116%	123%
Lateral Bending	INT	951	906
		100%	100%
	FUS-f-5c	1033	980
		109%	108%
	FUS-f-0c	1099	1062
		116%	117%
	FUS-5c	1019	958
		107%	106%
	FUS-0c	1078	1053
		113%	116%
Torsion	INT	314	345
		100%	100%
	FUS-f-5c	317	360
		101%	104%
	FUS-f-0c	325	399
		104%	116%
	FUS-5c	300	330
		96%	96%
	FUS-0c	320	374
		102%	108%

The percentages indicate the disc stresses of all models normalized by the disc stresses of INT

## Results

### ROM of FE models at all motion segments

**Fig. 2:**
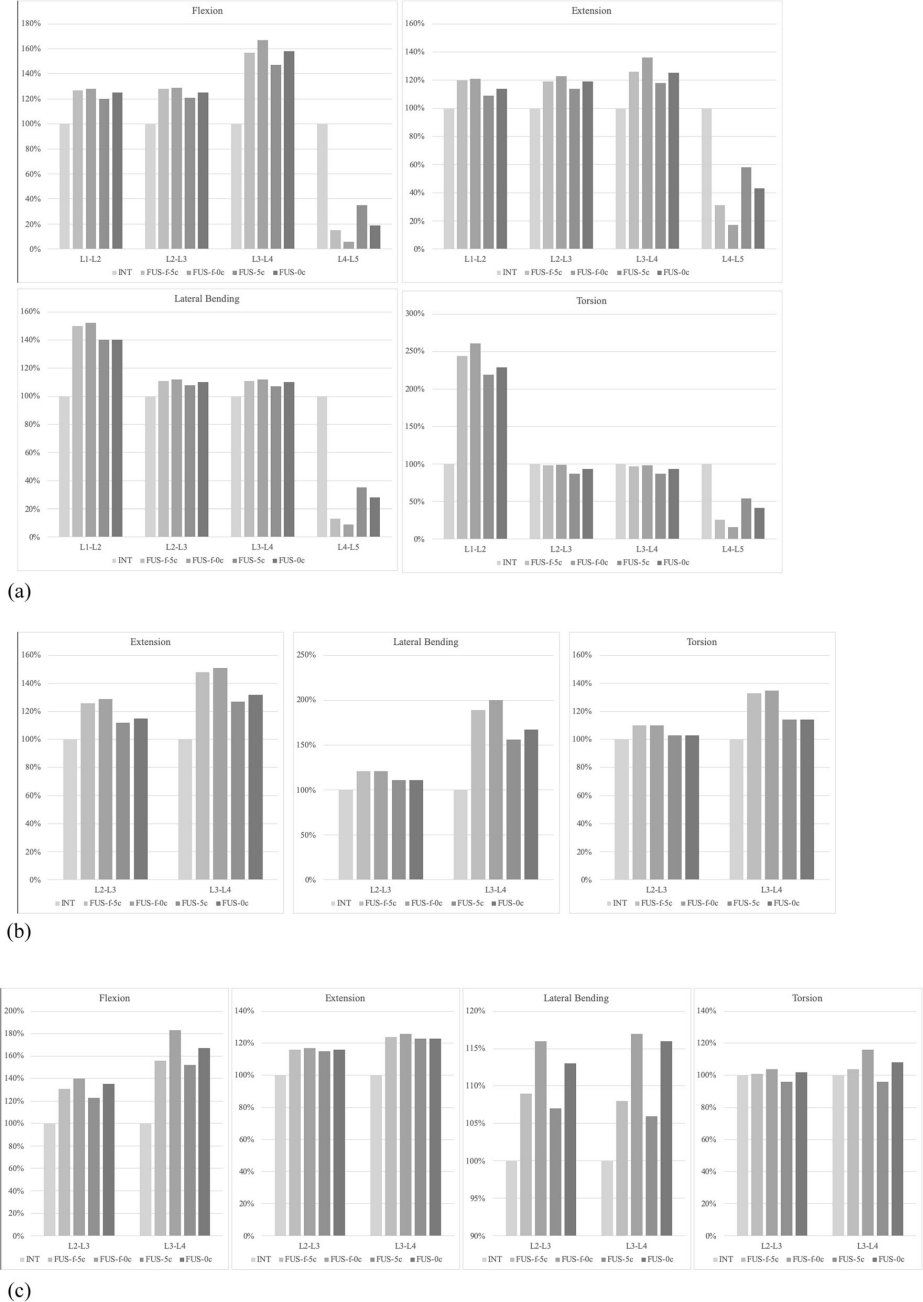
The **a**) range of motion (ROM), **b**) facet joint forces and **c**) disc stresses of all models normalized by the INT model

The range of motion of all FE models for all loading condition is summarized in Table 1 and Fig. 2a. The ROM of all implanted models was less than the intact model at the fusion segment but greater than the intact model at the adjacent segments. After fusion of L4–5 had finished and the fixator was removed, the ROM of the adjacent segments in the FUS-5c and FUS-0c models under all loading conditions was found to be similar to the intact (INT) spine.

### Facet joint forces in cephalic adjacent levels

The facet joint force (contact force) ratio was calculated as the ratio of facet joint force for each fusion model to the INT model. Table 2 and Fig. 2b detail the facet joint force ratio on the adjacent facet joint at the L2/L3 and L3/L4 level when the lumbar spine is placed under extension, lateral bending and torsion. In extension, the ratio at the L2/L3 facet was less than at the L3/L4 facet in all fusion models. The ratio at the adjacent facet joints in models with the fixator removed (FUS-5c and FUS-0c) was less than in the models with a fixator (FUS-f-5c and FUS-f-0c). The FUS-5c and FUS-0c models showed a similar facet joint force ratio at the adjacent facet joints. However, the facet joint force ratio at the adjacent facets declined after fusion had complete and the fixator was removed.

### The disc peak stresses at cephalic adjacent levels

The ratio of peak disc stress was calculated as the ratio from each fusion model to the INT model. Table 3 and Fig. 2c show the ratio of peak stress on the IVDs at the cephalic adjacent levels of L2/L3 and L3/L4 under flexion, extension, lateral bending and torsion. The peak disc stresses at the adjacent levels were significantly higher in all fusion models than in the INT model and, moreover, the disc stress ratio at the L3/L4 disc was greater than at the L2/L3 disc, except under lateral bending. Of all fusion models, the FUS-5c model had the lowest stress ratio. In flexion, the FUS-f-0c model showed the greatest change in peak disc stress at both L2/L3 and L3/L4, with the peak disc stress at L3/L4 increasing by 83%. Removing the fixator (FUS-5c and FUS-0c) resulted in a lower ratio of peak disc stress at the adjacent levels than situations where the fixator was retained (FUS-f-5c and FUS-f-0c).

## Discussion

Posterior lumbar interbody fusion with an internal fixation device is commonly used to stabilize an unstable lumbar spine after lumbar decompression surgery. Postoperative loss of lumbar lordosis has been reported in previous studies [15, 20], but the effects of changes in lumbar lordosis on adjacent segments and the use of internal fixation devices have not been widely investigated. The purpose of this study was to analyze the effects of postoperative biomechanical changes at adjacent segments following posterior lumbar interbody fusion with an internal fixation device. Finite element models were developed with different lordosis angles and analyzed before and after removal of the internal fixation device.

The results of this study show that the overall range of motion increased in cephalic adjacent levels for all fusion models (FUS-f-5c, FUS-5c, FUS-f-0c, FUS-0c). As the range of motion increased, this lead to changes in maximum von Mises stress on the disc and contact forces on the facet joints at cephalic adjacent levels. Implanting the cage with a neutral (0°) lordotic angle lead to adverse effects on the biomechanical conditions of cephalic adjacent levels; similar trends were reported by Zhao et al. [14]. Zhao et al. [14] created a lumbar model of L1-S5 and showed that both the range of motion and intradiscal pressure at adjacent segments increased after interbody fusion with pedicle screws, and a decrease in lordosis at the fusion site increased the range of motion and intradiscal pressure at adjacent segments in all motion conditions. This current study demonstrated major improvements in intradiscal pressure and facet contact force at adjacent segments after removal of the internal fixation device, especially in situations where there was a loss of lordosis following fusion.

In this study, the performance of the internal fixation device in terms of range of motion, maximum von Mises stresses at cephalic adjacent levels, and contact force on facet joints was investigated when the lumbar spine was implanted with an interbody cage to simulate 0° and 5° of lordosis. The implanted spines were subjected to flexion, extension, left torsion, and left lateral bending. The highest von Mises stresses at cephalic adjacent discs occurred in the FUS-f-0c model when placed under flexion. Increasing the angle of the interbody cage from 0° to 5° acted to reduce the maximum von Mises stresses at L3/L4 by 14.8, 1.7, 7.7 and 9.8% for flexion, extension, lateral bending, and torsion motions respectively; a loss in lordotic angle was found to have a relatively low impact on the L2/L3 intradiscal pressure. A decrease in lordosis at the instrumented level may accelerate adjacent segment diseases [7–9] as the center of gravity moves anteriorly, resulting in greater loading across the anterior column of lumbar spine. A cadaveric study by Umehara et al. [9] reported that increased loading was found not only at the adjacent disc but also on the internal fixation device when hypolordosis occurred at the instrumented level. This current study showed similar facet joint contact forces at adjacent segments in the FUS-f-5c and FUS-f-0c models, and the values were all higher than the intact model. These results demonstrated the influence of lordotic angle on the range of motion, intradiscal pressure, and facet joint contact force of the adjacent segments following spinal fusion. The aforementioned adverse effects of a loss in lordotic angle on the loading of the adjacent segment may cause degenerative changes at the segment nearest the fusion site, in accordance with reported long-term complications of lumbar fusion [13].

After removal of the internal fixation device, both the 0° and 5° lordotic models showed an increase in the range of motion at the fused segment and a decrease in intradiscal pressure and facet joint contact force on the adjacent segments. The stress is more equally distributed in adjacent segments after removal of the internal fixation devices, which may also help to reduce the incidence of adjacent segment diseases. Hsieh et al. [11] suggested that removing spinal fixators after complete fusion could reduce the incidence of adverse effects at adjacent segments. Similarly, Jeon et al. [21] indicated that removing the internal fixation instrument could alleviate pain and disability and improve the clinical and radiographic outcome.

In the FUS-5c model (fixator removed, cage angled at 5°) the maximum von Mises stresses at L3/L4 were 4, 1, 2 and 3% less in flexion, extension, lateral bending, and torsion than the condition before removal (FUS-f-5c). The increased mobility (elastic deformation) of the fused segment (L4/5) is likely the reason for the decrease in intradiscal pressure at the adjacent segment. The same trend was seen for the facet joint contact force, whereby the contact force at L3/L4 in the FUS-5c model was 21, 33, and 19% less for extension, lateral bending and torsion motions, respectively, in comparison to the FUS-f-5c model. As with the L3/L4 segment, the maximum von Mises stresses and facet joint contact forces at L2/L3 were lower than the interbody fusion model with the pedicle screw fixation system. The FUS-0c (fixator removed, cage angled at 0°) model had lower maximum von Mises stresses and facet joint contact forces at the adjacent segments than both the FUS-f-0c and FUS-f-5c models, signifying that the impact of lordotic loss at adjacent segments could be diminished by removal of the internal fixation device.

This study simulated single-level interbody fusion (L4/L5) by a mathematical model, while the interbody fusion of other levels was not analyzed. Our model produced solid predictions but which needs to be validated with a cadaver based biomechanical study or a clinical follow-up. This may limit the direct clinical applications that can be derived from the findings. Similarly, concomitant lordotic changes at adjacent segments after implantation of the cage were not considered. The properties of the spine were also simplified, as the structure of the vertebral body was assumed to be isotropic and homogenous. The models also did not account for the mechanical effects of muscle contraction. The models were simplified in this way because of the complexity of the spinal geometry, and the numerous material properties and boundary conditions that come into play during physiological loading. However, these simplifications do not detract from the findings of this study, as the models considered focus on a specific region of the spine and allow the cause-effect relationships to be isolated and fully explored.

## Conclusion

Maintaining the lordotic angle and removing the spinal fixator after complete fusion has occurred should be considered in order to reduce complications at the adjacent levels. This also acts to reduce the intradiscal pressure and facet contact forces at adjacent segments.

## Data Availability

The datasets used and/or analyzed during the current study are available from the corresponding author upon reasonable request.

## Abbreviations

FUS-0c: Lumbar spine after removal of the spinal fixator from the FUS-f-0c model
FUS-5c: Lumbar spine after removal of the spinal fixator
FUS-f-0c: Lumbar spine implanted with a non-lordotic intervertebral cage and spinal fixator at L4-L5
FUS-f-5c: Lumbar spine implanted with a lordotic intervertebral cage and spinal fixator at L4-L5
INT: Intact lumbar spine
IVD: Intervertebral disc; FCFs: facet contact forces
